# Brain dynamics and personality: a preliminary study

**DOI:** 10.3934/Neuroscience.2024030

**Published:** 2024-12-12

**Authors:** Francesco Ciaramella, Lorenzo Cipriano, Emahnuel Troisi Lopez, Arianna Polverino, Fabio Lucidi, Giuseppe Sorrentino, Laura Mandolesi, Pierpaolo Sorrentino

**Affiliations:** 1 Department of Motor and Wellness Sciences, University of Naples “Parthenope”, 80133 Naples, Italy; 2 NeapoliSanit Rehabilitation Center, Ottaviano (NA), 80044 Naples, Italy; 3 Department of Education and Sport Sciences, Pegaso Telematic University, 80143 Naples, Italy; 4 ICS Maugeri Hermitage Napoli, 80145 Naples, Italy; 5 Department of Social and Developmental Psychology, Faculty of Medicine and Psychology, University of Roma “Sapienza”, 00185 Rome, Italy; 6 Forensic Science and Social Governance Disciplinary Innovation Base of Zhongnan University of Economics and Law, 430073, Wuhan, China; 7 Department Humanities, University of Naples “Federico” II, 80133 Naples, Italy; 8 Department of Biomedical Sciences, University of Sassari, 07100 Sassari, Italy; 9 Institut de Neurosciences des Systèmes, Inserm, INS, Aix-Marseille University, 13005 Marseille, France

**Keywords:** brain flexibility, temperament and character inventory, neuronal avalanches, magnetoencephalography (MEG), cooperativeness

## Abstract

Personality can be considered a system characterized by complex dynamics that are extremely adaptive depending on continuous interactions with the environment and situations. The present preliminary study explores the dynamic interplay between brain flexibility and personality by taking the dynamic approach to personality into account, thereby drawing from Cloninger's psychobiological model. 46 healthy individuals were recruited, and their brain dynamics were assessed using magnetoencephalography (MEG) during the resting state. We identified brain activation patterns and measured brain flexibility by employing the theory of neuronal avalanches. Subsequent correlation analyses revealed a significant positive association between brain flexibility and cooperativeness, thus highlighting the role of brain reconfiguration tendencies in fostering openness, tolerance, and empathy towards others. Additionally, this preliminary finding suggests a neurobiological basis for adaptive social behaviors. Although the results are preliminary, this study provides initial insights into the intricate relationship between brain dynamics and personality, thus laying the groundwork for further research in this emerging field using a dynamic network analysis of the functional activity of the brain.

## 1. Introduction

The complex interaction between genetic and environmental factors induces changes in the brain that inevitably affect individuality and behavioral choices [1]. In the last decade, personality neuroscience has emerged as a new field of investigating personality and individual differences [2]–[4]. The goal is to understand the relationship between the brain and personality, mainly in the general population [5]. In this framework, personality is considered in relation to neurobiological correlates, and understanding brain functioning is the basis of knowledge of the characteristics regulating behavior and becomes a valid prerequisite for predicting it.

In a previous magnetoencephalography study, by taking the perspective of the brain as an interconnected network into account [6], we used Cloninger's psychobiological model to study how individual differences are correlated with specific cerebral structures [7]. Using the Temperament and Character Inventory (TCI) [8], this model identified four primary-basic personality temperaments (Novelty Seeking, NS; Harm Avoidance, HA; Reward Dependence, RD; and Persistence, P) and three characters (Self-Directedness, SD; Cooperativeness, CO; and Self-Transcendence, ST). In particular, during a resting state condition, we observed high HA scores that were associated with a reduced centrality of the left caudate nucleus, which suggested that this nucleus could play a key role in adaptive behavior, probably for its involvement in the insular salience network [7].

Other studies have focused on understanding the relationships between personality and neurobiological correlates using different theoretical constructs such as the Big Five model/five-factor model [9],[10]. The correlation between personality dimensions and specific cerebral networks has been highlighted, thereby evidencing changes in the volumes of frontal, temporal, and parietal areas as well as of the insula, amygdala, and basal ganglia [11]–[16]. For example, Aghajani and colleagues [17] evidenced specific functional connectivity patterns originating from the amygdala that correlated to neuroticism and extraversion scores. Additionally, the role of the cerebellum in sustaining motivational temperamental traits has been documented [18]–[20], thus confirming the insight of the cerebellum on social and affective behaviors [21],[22].

To date, most studies dealing with brain connectivity and personality have examined the interplay among regions using time-averaged data (static networks), while only a few have explored the time-evolving interaction (among multiple brain areas) that rapidly changes in response to external and internal demands (dynamic networks). The human brain works in a critical regime [23],[24]; to adapt to the surrounding and changing environment, the brain performs continuous and efficient reconfigurations of patterns of activity that reflect the coordinated recruitment of different areas in any circumstance [25],[26]. The more the brain reconfigures itself (i.e., the more flexible), the more appropriate its reply will be to the environmental stimulus [27]–[29]. This adaptability is mirrored in large-scale bursts of activations in the brain signals, called “neuronal avalanches” [30], which repeatedly remodel the brain functional connectome, giving rise to a large number of patterns (spatio-temporal sequences of regions that are recruited in avalanches) over time. The number of such patterns (i.e., the number of avalanche configurations) is named “functional repertoire” and provides a measure of brain flexibility. The brain propensity to reconfigure itself has been related to several aspects involved in personality architecture, such as executive function, creativity, and resilience [31]–[34], thus suggesting a role of brain network dynamics in psychological outcomes.

Recently, personality has been considered a system characterized by complex dynamics that are extremely adaptive depending on continuous interactions with the environment and situations [35]. Therefore, in this context, a dynamic network analysis of the functional activity of the brain proves to be a useful approach to studying personality.

In this work, we hypothesize that the features of brain dynamics may underlie specific personality characteristics. To verify this hypothesis, we first acquired 46 healthy people by a magnetoencephalography (MEG) system in order to estimate the brain dynamic patterns during resting state. Although it is known that personality studies typically involve larger sample sizes, the use of MEG in this study partly justifies the choice of a smaller sample. However, this constitutes a limitation of this study. Specifically, starting from the reconstructed time series of the brain signals, we identified a *neuronal avalanche*, which is defined as an event that begins when at least one brain region deviates from its baseline activity and ends when all regions restore their typical level of activity. To identify the avalanches, each source-reconstructed signal was z-scored over time and then dichotomized to 1 (one) if it was above the definite threshold and to 0 (zero) otherwise. Given a neuronal avalanche, we defined its corresponding *pattern* as the set of all the brain areas that were recruited. Subsequently, we defined the *functional repertoire* as the set of unique (excluding repetitions) patterns that happened over time and used its size as a marker of brain flexibility. Second, we used the Temperament and Character Inventory (TCI)questionnaire to investigate the cohort's personality features. Finally, we investigated the relationship between personality dimensions, which was assessed by the TCI, and brain flexibility estimated as the size of the functional repertoire by a correlation analysis.

## 2. Material and methods

### 2.1. Participants

Data from forty-six healthy subjects (27 females, 19 males, mean age: 27.6 ± 4.7 years) were included in the study. The dataset partially overlaps with the one presented by Troisi Lopez et al. [7]. The exclusion criteria included the following: age > 40 years, left-handedness, personal history of neurological, psychiatric, or psychological illness, and psychoactive drug use. All of the participants provided written informed consent. The study was conducted in accordance with the Declaration of Helsinki and was approved by the Local Ethics Committee of the University of Naples “Federico II” (Prot. no. 17/2021).

### 2.2. The Temperament and Character Inventory (TCI)

The participants completed the TCI consisting of 240 items true/false questionnaire [8]. TCI is a self-rated instrument that provides a comprehensive inventory of dimensions of temperament (Novelty Seeking, NS; Harm Avoidance, HA; Reward Dependence, RD; and Persistence, P) and character (Self-directedness, SD; Cooperativeness, C; and Self-transcendence, ST). In general, NS refers to the tendency to seek novel stimuli and experiences; HA refers to a tendency to inhibition of behavior in response to signals of punishment; RD refers to a tendency to the maintenance of behavior in response to cues of social reward; and P measures the tendency towards perseverance in the face of adversity. In sum, temperament refers to the set of responses to emotional stimuli: it is native, it manifests from birth, and it modifies throughout life. For the concerns the three domains of character, SD measures the ability to use the willpower necessary to achieve personal goals; C measures the ability to cooperate with others; and ST measures the ability to look beyond self-interest to see oneself as part of a larger whole, which could be described as a marker of maturity, but could also be described as a tendency towards spirituality or a belief in religious figures or the supernatural [7].

The character is influenced both by sociocultural experiences and the path of individual maturation [8]. All these aspects of personality interact with each other, thus influencing the management of one's emotionality and behavioral choices.

**Table 1.** TCI assessment results of the sample in relation to each temperament and character.

	NS	HA	RD	P	S	C	ST
Mean	0,460	0,440	0,649	0,652	0,717	0,807	0,393
S.D.	0,164027	0,192991	0,167279	0,256733	0,142509	0,085748	0,181701
Min	0,150	0,029	0,208	0,125	0,295	0,643	0,121
Max	0,850	0,943	0,958	1,000	0,955	0,952	0,848

### 2.3. MRI acquisition

For the acquisition of the magnetic resonance (MR) images, a 1.5-T scanner equipped with an 8-channel parallel coil head (General Electric Healthcare, Milwaukee, WI, USA) was utilized either after the MEG recording or a minimum of three weeks later (but not more than one month). Three-dimensional T1-weighted images (gradient-echo Inversion Recovery sequence prepared Fast Spoiled Gradient Recalled-echo, repetition time = 6988 ms, TI = 1100 ms, TE = 3.9 ms, flip angle = 10, voxel size = 1 × 1 × 1.2 mm^3^) were acquired.

### 2.4. MEG acquisition

MEG data collection took place within a system consisting of 163 magnetometers located in a magnetically shielded room (AtB Biomag UG, Ulm, Germany). Before data acquisition, the position of the four reference points (i.e., nasion, right and left pre-auricular, and apex) were digitized using the Fastrak system (Polhemus). The participants were instructed to maintain closed eyes and a relaxed state (resting-state condition) during two separate recording sessions, each lasting 3.5 minutes. The instructions were relayed via an intercom immediately preceding each recording session. After data acquisition, an anti-aliasing filter was applied, and sampling occurred at 1024 Hz. Subsequently, a smoothing filter was employed to eliminate components below 0.5 and above 48 Hz. This filtering process was conducted offline using MatLab scripts within the Fieldtrip 201456 toolbox. Concurrently, electrocardiogram (ECG) and electrooculogram (EOG) signals were also registered.

### 2.5. Preprocessing

To minimize the influence of environmental magnetic interference, we conducted a principal component analysis (PCA) using the resources provided by the Fieldtrip Toolbox on cerebral magnetic signals. This involved orthogonalizing the reference signals to establish a basis and then projecting the brain sensor signals onto this noise basis. Subsequently, we eliminated these projections to yield the purified data. An expert evaluator visually assessed the entire dataset, identifying and excluding noisy acquisition segments. Additionally, an independent component analysis (ICA) was employed to eliminate cardiac (usually 1–2 components) and ocular (0–1 components) artifacts from the raw brain signals. Several noisy recording segments were consequently discarded based on the inspection results, thus leading to the division of the recording into multiple trials. To ensure reliable connectivity estimates, only trials that exceeded 4 seconds in duration were considered for the analysis.

### 2.6. Source reconstruction

We conducted a channel data source reconstruction by utilizing the beamforming technique integrated into the Fieldtrip toolbox [36]. Initially, subject fiducial points were employed to co-register the MEG data with the individual-specific MRI. Subsequently, by employing a single-shell volume conduction model and an equivalent current dipole source model, we applied a Linearly Constrained Minimum Variance (LCMV) beamformer to the entire pre-processed, wideband data [37]. This process aimed to reconstruct the time series data relative to the centroids of 116 regions of interest (ROIs), as delineated by the Automated Anatomical Labeling (AAL) atlas [38],[39]. Both the atlas and MRI were aligned with the head coordinates. We focused on the initial 90 ROIs and excluded those within the cerebellum due to the unreliable signal reconstruction in that region. A singular value decomposition (SVD) was utilized to project the time series along the direction of the dipole that explained the majority of the variance for each source. Subsequently, we visually assessed the source space data for each subject to ensure the absence of residual artifacts at the source level.

### 2.7. Analysis of dynamics

#### 2.7.1. Neuronal avalanche and avalanche configuration

The term “neuronal avalanche” denotes an occurrence characterized by substantial fluctuations in activity. To measure these fluctuations, we subjected each of the 90 reconstructed source signals to z-transformation, and subsequently applied a threshold of 3 standard deviations (i.e., z = 3) to each time series. Following this criterion, a neuronal avalanche commences when at least one region becomes active (i.e., surpasses the threshold of |z| > 3) and persists as long as any region remains above the threshold. It's worth noting that the spatial positions of the regions are not considered. Therefore, two coactive areas (temporally) are not necessarily adjacent and may also be situated in different and distant regions. However, the outcomes were not strictly influenced by the selection of this threshold. We conducted the analyses again, thereby setting the threshold at z = 2.75 and z = 3.25, and obtained similar results.

These analyses necessitate binning the time series. To determine an appropriate bin length, we calculated the branching ratio [40], σ, as follows: for each time bin duration, for each subject, for each avalanche, the geometrically averaged ratio of the number of events (activations) between the subsequent time bin and the current time bin was computed as follows:

i= 1Nbin-1j=1Nbin-1nevents j+1nevents j1Nbin-1

where σi is the branching parameter of the i-th avalanche in the dataset, Nbin is the total number of bins in the i-th avalanche, and nevents(j) is the total number of events in the j-th bin. Then, we (geometrically) averaged the results over all avalanches [41] as follows:

σ= 1Navali=1Navali1Naval

where Naval is the total number of avalanches in each participant's dataset. In branching processes, a branching ratio of σ  = 1 indicates critical processes with activity that is highly variable and nearly sustained, σ < 1 indicates subcritical processes in which the activity quickly dies out, and σ > 1 indicates supercritical processes in which the activity increases. The bin length equal to three samples yielded a critical process with σ = 1, thus justifying the use of the term “avalanche” for these events. This means that each bin is obtained from three time-points of the binarized time-series. However, the robustness of the results to changes in this exact bin length was also investigated, and the main results were not affected. That is, different binnings yielded branching ratios very close to one, and the statistical differences in the exploration of the functional repertoire were similar for each binning. For each avalanche, an “*avalanche configuration*” was defined as the set of all areas that were above the threshold at some point during the avalanche. Note that the definition of avalanche configuration implies a loss of information in terms of the temporal structure within each avalanche. After identifying avalanches, the avalanche configurations were computed by an automated MATLAB script that recognizes which brain regions were involved in each avalanche and saves the specific configurations.

### 2.8. Functional repertoire

We evaluated each participant's functional repertoire, which we define as the count of unique avalanche configurations expressed during the recording. By “unique”, we mean that each pattern of avalanche is tallied only once towards the total size of the functional repertoire. Indeed, since we are interested in assessing brain flexibility by means of the number of different configurations that the brain can present (i.e., the functional repertoire), even though many avalanches with the same configuration occur, we will count only one configuration, as it is identical across those avalanches. To ensure fairness in the comparisons among the participants, we considered the duration of the data acquisition, as it could influence the estimation of the functional repertoire. Consequently, the analysis was conducted using an equal amount of data for each participant (approximately 6 minutes). To achieve this, we measured the duration of each recording and identified the lowest one. Then, an automated script in MATLAB extracted a segment of data of the pre-identified lowest duration from each participant's recording, starting from a randomly selected time-point.

### 2.9. Statistical analysis

Analyses were performed using MATLAB (MathWorks, version R2013a). The functional repertoire was correlated to the three TCI characters and four temperaments scores using Spearman's correlation. A significance level of 0.05 was applied after false discovery rate (FDR) correction across correlation with temperaments/characters.

## 3. Results

The brain critical state observation presupposes the calculation of the branching ratio of the time series binned at different lengths. For a threshold of z = 3, bin 1 results showed a branching ratio σ = 1. Hence, for the time series of bin 1, we calculated the number of visited patterns. Subsequently, a Spearman's correlation analysis was performed to estimate the relationship between the dynamic brain parameter and both the three dimensions of character and the four of temperament.

**Figure 1.**
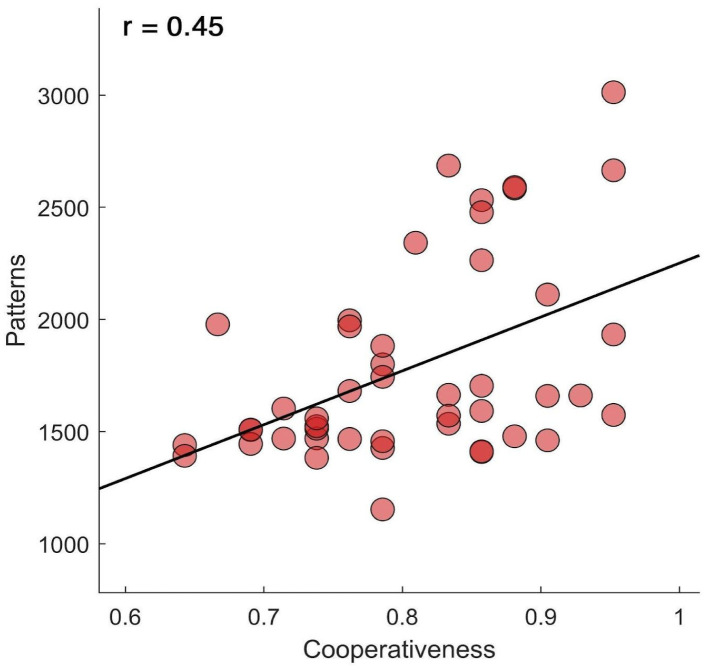
Correlation between the number of avalanche patterns and TCI. The graph represents the Spearman's positive correlation between the number of visited patterns of configuration in broadband and the cooperativeness, one of the three TCI characters (r = 0.45, p = 0.002, pFDR = 0.012).

As reported in Figure 1, the results show a statistically significant correlation between the number of visited patterns of configuration in broadband and the cooperativeness, which is one of the three TCI characters (r = 0.45, p = 0.002, pFDR = 0.012). We did not find further significant results in the correlation analysis. In addition, in order to check our results, the correlations were repeated by modifying the bins (bin = 2, pFDR = 0.005, r = 0.449; bin = 3, pFDR = 0.007, r = 0.434; bin = 4, pFDR = 0.007, r=0.434; bin = 5, pFDR = 0.009, r = 0.426) and the thresholds (thresholds 2.75, pFDR = 0.004 r = 0.458; thresholds 3.25, pFDR = 0.004, r = 0.456). The results confirmed the correlation.

## 4. Discussion and conclusion

In this preliminary study, we set out to investigate whether brain dynamics features may underlie specific personality dimensions. In particular, we recruited forty-six healthy individuals and investigated their brain dynamics activity by means of MEG. Then, we applied the theory of neuronal avalanches to identify activation patterns of brain regions and to measure brain flexibility. Subsequently, we investigated potential correlations between brain flexibility and the Cloninger model dimensions of personality, which revealed a significant positive correlation with the character of cooperativeness (C). This preliminary finding suggests that the higher an individual's reconfiguration of brain states, the higher the inclination for openness, tolerance, and, ideally, empathy toward others.

By applying the neuronal avalanche theory, the analysis of brain flexibility used in this study overcame the limitations of previous studies that analyzed the biological correlates of personality dimensions without taking the continuous remodeling changes of the brain's functional connectome into account. In other words, our analysis of “functional repertoire” allowed us to obtain a dynamic measure of brain flexibility and, consequently, to study the personality characteristics with an innovative approach. In this case, we can interpret the data obtained by considering the dynamic nature of the psychological dimension of cooperativeness as the expression of a brain capable of effectively readjusting. As an example, we can report our previous MEG study conducted on a partially overlapping sample, in which we applied the network theory to evaluate the relation between the brain network topology and the personality dimensions [7]. In this study, we showed a negative correlation between the left caudate nucleus and the harm avoidance temperament, thus suggesting that this nucleus is involved in adaptive behavioral responses and in choosing actions that are likely to lead to a positive outcome [42]. Although the data obtained is interesting, the previous study did not allow us to evaluate the brain dynamics or analyze the relationship between continuous brain readjustments and the personality dimensions that are most affected by continuous situational changes. Indeed, brain flexibility encompasses the brain's ability to swiftly reconfigure itself in response to various environmental stimuli, thereby effectively encoding information. In social contexts, individuals engage with others, thereby communicating through verbal, paraverbal, and nonverbal means. The information involved in such interactions is highly complex, and effective communication between brain regions is essential to understand social cues, empathizing with others, and coordinating actions [43]. A flexible reconfiguration of brain patterns may enable the rapid integration of information from various sources, thus contributing to nuanced social responses. Additionally, considering that multiple individuals can participate in social exchanges and that an individual's attention is divided among various aspects (e.g., observing the environment, attending to physiological needs, situating oneself temporally), the cognitive workload becomes immensely substantial. In such instances, a high level of brain flexibility may allow one to effectively manage this multitasking task [44]. Consequently, an individual with a high degree of brain flexibility might find enjoyment in social interactions as they adeptly manage the situation and feel at ease. This propensity could correspondingly manifest as a high score in cooperativeness. Conversely, an individual with a low brain flexibility might struggle to rapidly process and manage the abundance of information present in socially charged situations, thus leading to difficulties and discomfort within the context [43]. Similarly, this might correlate with a low score in cooperativeness, which is in line with the aforementioned points.

To our knowledge, few studies on the dynamics of the brain and personality dimensions have been conducted so far. Most of these studies have assessed personality using the five factor model (FFM), thereby showing that individual differences in conscientiousness traits are mediated by specific functional dynamics on a large scale [45] (Toschi et al. 201). Furthermore, Kabbara and colleagues [46] found that extraversion and openness are positively correlated with brain network dynamics. Similarly, Riccelli et al. showed that openness is associated with several brain regions, including parietal, temporal, and frontal areas, which are implicated in a wide range of sociocognitive functions [47]. This finding shows that the contribution of different regions from the whole brain network may be in line with our finding, which considers cooperativeness related to the reconfiguration of the brain system on a large scale. Cooperativeness could be associated with the trait of extroversion, as they share similar characteristics in social behaviors. In fact, a comparative study between FFM and TCI showed a relationship between the cooperativeness of TCI and the openness and extroversion of FFM [48]. From this point of view, greater cooperativeness and less egocentric and hostile behaviors can result from a flexible brain that modulates its activity by establishing flexible and adaptive interactions with its environment. It must be highlighted that the psychobiological model proposed by Cloninger takes the influence that learning and sociocultural factors exert on personality traits into great consideration, especially characters, since interactions between the human social system and the surrounding ecosystem strongly influence the latter. For this reason, it has been proposed to take the role of individual sociocultural experiences in mediating the relationship between brain dynamics and personality into consideration [29].

In agreement with the dynamic approach to personality and taking the relation between brain flexibility and prosocial behaviors into account, personality and individual differences cannot be understood without understanding the dynamic processes of brain reconfiguration.

Our preliminary study does not come without some limitations. First and foremost, it is important to note the limited size of our sample compared to traditional personality studies, which does not allow for intra-group analyses. However, it should be remembered that this is a preliminary study based on MEG data, and similar studies often report comparable sample sizes [46]. In this regard, it should be underlined that our sample is very homogeneous compared to other personality studies, thus allowing for a precise snapshot of personality dynamics in a specific age range. Another limitation is the exclusion of the cerebellum from the analysis of brain dynamics because our MEG system did not cover the lower occipital part and, therefore, did not allow a clean recording of the signal coming from the cerebellar nuclei. It is essential to note this limit in light of recent studies that reveal the cerebellar role in modulating individual differences in approach and avoidance behaviors by means of a cortico-basal-cerebellar loop [18],[19]. Furthermore, the choice to use the TCI instead of other personality questionnaires may also constitute a limit in the generalization of our results. Further studies, with a greater sample and a follow-up to verify the stability of the results, as well as the use of multimodal neuroimaging techniques or indicators to provide more comprehensive information about brain activity, will be necessary to confirm the relationship between personality dimensions and brain flexibility.

Despite these limitations, the present preliminary study could be considered the first contribution to explain the neurobiological aspects of the dynamic system of personality, and it is part of an innovative line of research that underscores the potential of a network-based perspective to enhance understanding of brain-behavior relationships [49]–[51].
